# Community-level antibiotic access and use (ABACUS) in low- and middle-income countries: Finding targets for social interventions to improve appropriate antimicrobial use – an observational multi-centre study

**DOI:** 10.12688/wellcomeopenres.11985.1

**Published:** 2017-07-28

**Authors:** Heiman F.L. Wertheim, Nguyen Thi Kim Chuc, Sureeporn Punpuing, Wasif Ali Khan, Margaret Gyapong, Kwaku Poku Asante, Khatia Munguambe, F. Xavier Gómez-Olivé, Proochista Ariana, Johannes John-Langba, Betuel Sigauque, Tran Khanh Toan, Stephen Tollman, Amelieke J.H. Cremers, Nga T.T. Do, Behzad Nadjm, H. Rogier van Doorn, John Kinsman, Osman Sankoh

**Affiliations:** 1 Nuffield Department of Clinical Medicine, University of Oxford, Oxford, OX3 7BN, UK; 2 Oxford University Clinical Research Unit, Hanoi, Vietnam; 3 Department of Medical Microbiology and Radboud Centre for Infectious Disease, Radboud University Nijmegen Medical Centre, Nijmegen, 6525, Netherlands; 4 Hanoi Medical University, Hanoi, Vietnam; 5 INDEPTH Network, Accra, Ghana; 6 Kanchanaburi HDSS, Institute for Population and Social Research, Mahidol University, Salaya, 73170, Thailand; 7 International Centre for Diarrhoeal Disease Research, Bangladesh, Dhaka, 1000, Bangladesh; 8 Dodowa INDEPTH Site, Dodowa, Ghana; 9 University of Health and Allied Sciences, Ho, Ghana; 10 Kintampo INDEPTH Site, Kintampo, Ghana; 11 Manhica Health Research Site, Manhica, Mozambique; 12 MRC/Wits Rural Public Health and Health Transitions Research Unit (Agincourt), University of the Witwatersrand, Park Town, Johannesburg, 2193, South Africa; 13 University of Kwazulu-Natal, Durban, 4041, South Africa; 14 Department of Public Health and Clinical Medicine, Epidemiology and Global Health, Umeå University, Umeå, SE-901 87, Sweden; 15 School of Public Health, Faculty of Health Sciences, University of the Witwatersrand, Parktown, Johannesburg, 2193, South Africa; 16 Department of Mathematics and Statistics, Njala University, Njala, Sierra Leone

**Keywords:** antibiotic, provision, access, use, low and middle income, community

## Abstract

In many low- and middle-income countries (LMICs), a poor link between antibiotic policies and practices exists. Numerous contextual factors may influence the degree of antibiotic access, appropriateness of antibiotic provision, and actual use in communities. Therefore, improving appropriateness of antibiotic use in different communities in LMICs probably requires interventions tailored to the setting of interest, accounting for cultural context. Here we present the ABACUS study (AntiBiotic ACcess and USe), which employs a unique approach and infrastructure, enabling quantitative validation, contextualization of determinants, and cross-continent comparisons of antibiotic access and use. The community infrastructure for this study is the INDEPTH-Network (International Network for the Demographic Evaluation of Populations and Their Health in Developing Countries), which facilitates health and population research through an established health and demographic surveillance system. After an initial round of formative qualitative research with community members and antibiotic suppliers in three African and three Asian countries, household surveys will assess the appropriateness of antibiotic access, provision and use. Results from this sample will be validated against a systematically conducted inventory of suppliers. All potential antibiotic suppliers will be mapped and characterized. Subsequently, their supply of antibiotics to the community will be measured through customer exit interviews, which tend to be more reliable than bulk purchase or sales data. Discrepancies identified between reported and observed antibiotic practices will be investigated in further qualitative interviews. Amartya Sen’s Capability Approach will be employed to identify the conversion factors that determine whether or not, and the extent to which appropriate provision of antibiotics may lead to appropriate access and use of antibiotics. Currently, the study is ongoing and expected to conclude by 2019. ABACUS will provide important new insights into antibiotic practices in LMICs to inform social interventions aimed at promoting optimal antibiotic use, thereby preserving antibiotic effectiveness.

## Introduction

Antibiotic resistance makes global dedication to facilitate the appropriate use of antibiotics an imperative. Two recent reports on antibiotic resistance have illustrated the importance of conducting studies in low- and middle-income countries (LMICs) where local data are scarce and the problem is significant ^1, 2^. Improving the appropriate use of antibiotics necessitates understanding their supply, as well as the social and cultural factors that create demand in the community. As different LMIC settings show distinct rates of over-the-counter antibiotic dispensing ^3, 4^, it is clear that the social, cultural and policy-related determinants of antibiotic practices are context-specific.

**Figure 1.**
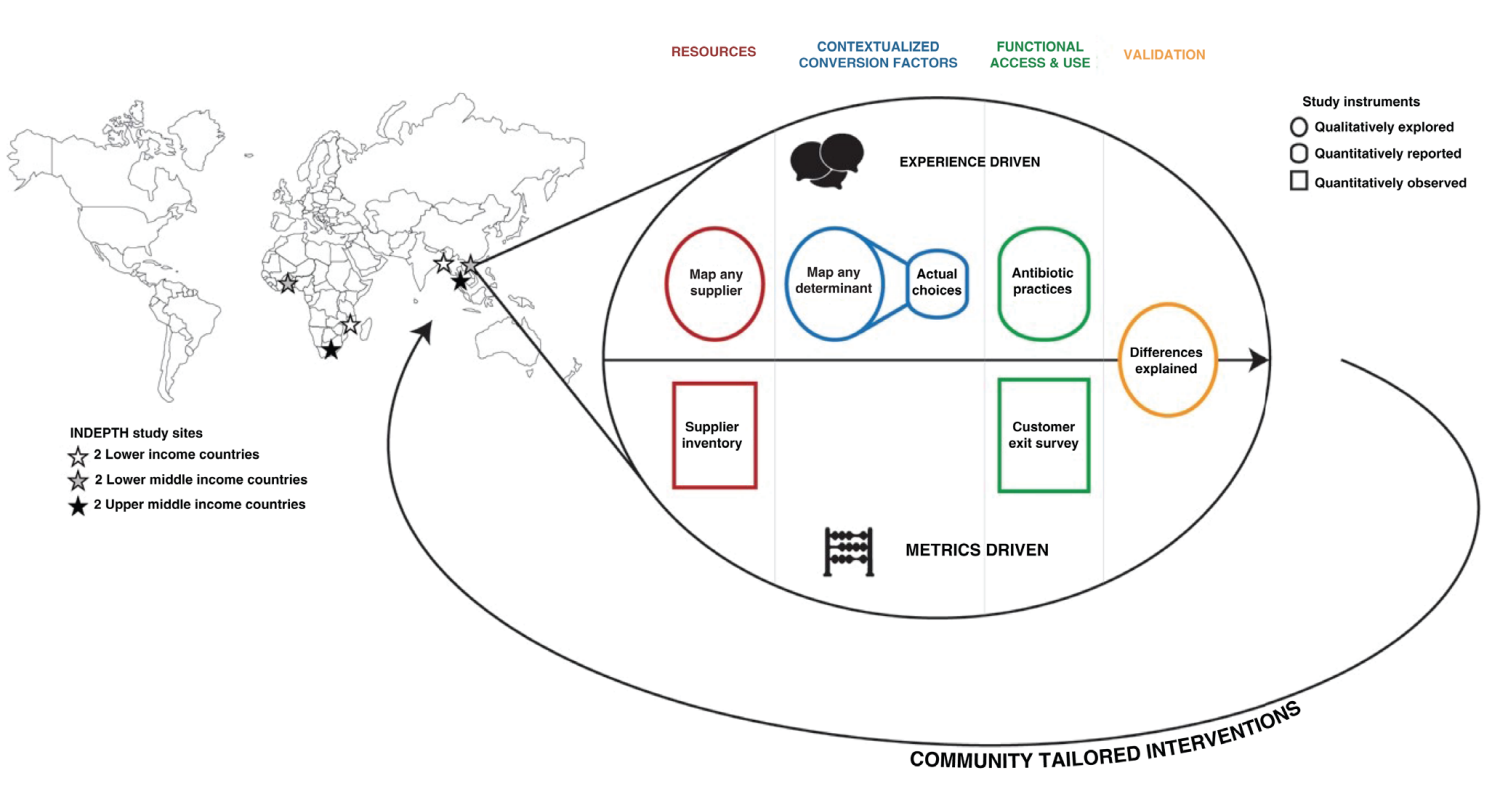
Study overview, including study location.

The aim of this project is to compare community-based antibiotic access and consumption practices across communities in LMICs in Asia (Bangladesh, Thailand and Vietnam) and Africa (Mozambique, Ghana and Republic of South Africa), in order to inform the design of, and identify targets for community-based intervention strategies that may be used to promote appropriate antibiotic use. Our hypothesis is that both antibiotic practices as well as their determinants differ across LMICs, and therefore warrant tailored intervention strategies. To examine this hypothesis, we will compare antibiotic access and use in the community between six different LMICs, and identify their main drivers through both qualitative and quantitative measures. A core asset of this study is that it covers multiple aspects involved in understanding community antibiotic practices: supply and demand; reported and observed practices; and local to cross-continental comparison ( Figure 1).

## Rationale

The study will be conducted in seven existing health and demographic surveillance system sites in six LMICs (Bangladesh: Matlab; Thailand: Kanchanaburi; Vietnam: Filabavi; Mozambique: Manhica; Ghana: Dodowa and Kintampo; Republic of South Africa: Agincourt) that are part of the INDEPTH-Network ( http://www.indepth-network.org/member-centres). INDEPTH is a global network of Health and Demographic Surveillance Sites (HDSSs), with 45 member centres and 52 HDSS field sites in 20 LMICs in Africa, Asia and Oceania. We will first perform in-depth qualitative interviews and focus group discussions (FGDs) in each participating site to explore the prevailing factors that affect antibiotic practices at each study site. The interviewees will include both antibiotics suppliers and consumers. This qualitative information will facilitate the development of a longitudinal quantitative household survey. This will then be followed by another set of in-depth interviews and FGDs with the aim of explaining any potential discrepancies between reported and observed use of antibiotics within each study site. The supply of antibiotics to the communities involved will be determined by standardized drug outlet inventories and customer exit interviews. The gained insight into antibiotic practices in different LMICs will inform the design of tailored intervention strategies to promote appropriate antibiotic use in the different settings. In addition, this project provides a uniform framework for appraising current antibiotic use patterns, which may subsequently be used in other LMIC communities.

The proposed study to investigate community antibiotic use in six LMICs is informed by Amartya Sen's Capability Approach (CA) ^5^. The CA describes individuals as having an endowment of resources that determines their capabilities: what they are able to be and do. The capability set comprises all of the things a person is able to be and do given their resource endowment. The individual has the choice to act on those capabilities to achieve certain states of being and doing, or what Sen terms ‘functionings’.

In our case, the resource of interest is the appropriate provision of antibiotics. However, we recognize that simply having appropriate provision does not necessarily translate into appropriate use. There are a number of factors, ranging from individual characteristics, to broader economic constraints, cultural norms, social frameworks, and political structures that can influence how antibiotics can be used. In Sen's terms, these are the 'conversion factors' that enable a possible set of outcomes (what he terms as 'capabilities'). Operationalising the CA is most suitably done by employing both qualitative and quantitative approaches ^6^. Qualitatively exploring individual values and contextual constraints is elemental to the framework.

## Study design

**Figure 2.**
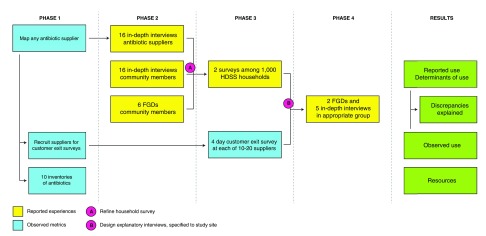
Overview of study design at each of six study sites. HDSS: Health and Demographic Surveillance Systems; FGD: focus group discussion.

This is an observational multicentre study that implements a set of interviews among drug suppliers and community members across six LMICs, which will be performed consecutively over a 2.5-year study period. The study consists of two major components that are based on ‘reported experiences’ and ‘observed metrics’, respectively ( Figure 2).

Initially, antibiotic resources will be ascertained by mapping all antibiotic suppliers ( Supplementary File 1) and by performing inventories of the antibiotics supplied ( Supplementary File 2). In the second phase of the study, factors affecting community antibiotic access and use that prevail in each study site will be explored through preparatory qualitative in-depth interviews ( Supplementary File 3 and Supplementary File 5) and FGDs ( Supplementary File 4 and Supplementary File 6). The results will be used to refine the subsequent quantitative longitudinal HDSS household surveys ( Supplementary File 7) conducted in Phase 3, which will entail two contacts with each of 1,100 participating households over a 1-year time period. Simultaneously, antibiotic supply to the community will be quantified through standardized customer exit interviews ( Supplementary File 8), which will be conducted on four days per selected supplier spread out over a one-year study period. Finally, to explain any potential discrepancies between results from the household survey and the customer exit survey, another round of qualitative in-depth interviews and FGDs will be performed.

## Study population

### Study sites

The project will take place in rural communities of six countries in Africa and Asia that have been chosen based on their income status in 2013. Using the World Bank classification of LMIC (criteria for 2014) ( https://datahelpdesk.worldbank.org/knowledgebase/articles/906519), we selected: Bangladesh and Mozambique representing lower income countries (LIC), Vietnam and Ghana representing lower-middle income countries (MIC-L), and Thailand and South Africa representing upper middle income countries (MIC-U). To facilitate the study, the chosen countries all contain at least one INDEPTH community-based study site. INDEPTH can provide a readily available and relevant sampling frame, and is therefore uniquely positioned to answer pressing questions on health in community settings. Globally, the INDEPTH Network surveillance sites study the life events of approximately 3 million people through on-going demographic surveillance. The network was set up in 1998 and has developed tools to measure, map and track the socio-demographic impact of cause-specific morbidity and mortality in LMIC populations using comparable methodologies in different countries ^7, 8^.

### Sample selection process

***Antibiotic suppliers.*** For each study site, all possible purchase or dispensing points for antibiotics (antibiotic suppliers) will be mapped and are eligible to participate in this study. Potential dispensing points will be identified based on both official lists provided by local authorities and local knowledge of the HDSS sites. This includes any formal or informal antibiotic supplier: from public hospital pharmacy to street vendor. The expected number of daily antibiotic encounters (a customer encounter that involves supply of antibiotics), and the proportions of the different types of suppliers will be determined from the mapping exercise.

Antibiotic suppliers 18 years old and older are eligible for three of the study elements: the customer exit survey, the inventory of antibiotic resources at vendor level, and the qualitative in-depth interview.

In each study site, 20 suppliers will be selected for customer exit interviews. The antibiotic suppliers will be recruited according to highest rank in terms of the number of daily antibiotic encounters.

The selection process of 10 antibiotic suppliers for inventories and 16 antibiotic suppliers for exploratory in-depth interviews will ensure similar proportions of the supplier types as identified in the mapping exercise in the corresponding study site. The HDSS field workers will characterized all identified antibiotic suppliers and will calculated the proportions of different types of antibiotic suppliers across the study sites. The suppliers will be randomly selected using the Excel random number function. The randomly selected suppliers will be approached sequentially to get permission to participate in these procedures until the required number is reached. 

For the qualitative in-depth interviews, employees who interact with customers will be included. Ideally, each in-depth interviewee will be affiliated with a unique antibiotic supplier, but if fewer than 16 eligible antibiotic suppliers can be identified in each site, a maximum of two employees from the same business may participate in the in-depth interviews.

Any supplier who does not consent for participation in a particular study element, will still be eligible for inclusion in the other study elements.

***Community members for qualitative in-depth interview.*** Community members 18 years-old and older who are willing to speak about their experiences with, and their attitudes towards medicines, are eligible to participate in this study. Different target populations from the community will be included in the initial round of exploratory in-depth interviews. From the HDSS-database, we will select eight mothers who care for children under 5 years old. The other eight participants should not be mothers of children under 5 years old, and they will include two males and two females 18 to 60 years old, plus two males and two females above 60 years old. These participants will be randomly selected against above inclusion criteria. The rationale for this inclusion criteria is that involving subjects from different age categories and gender will assure achieving a more informative view. In addition, children under five years-old and adults above 60 years-old have a higher frequency of healthcare seeking. Household female members, particularly mothers, play a crucial role in managing childhood and family illness, thus they will predominantly be selected. In this study, we will avoid having two participants from the same household ( Table 1). Community members who participate in a qualitative in-depth interview will be excluded from the exit interview and FGD. Any community member who does not consent to participate in a qualitative in-depth interview will still be eligible for inclusion in an FGD. If the final explanatory in-depth interviews (Phase 4) require participants from outside the groups mentioned above, the inclusion criteria will be redefined for that site.

**Table 1.** Stratification of focus group discussion.

Focus group category	Geographical area within the HDSS ^*^	Preparatory FGDs in phase 2 (number of participants)
Females ≥18 and <30 years old	A	1 (6-8)
Males ≥18 and <30 years old	B	1 (6-8)
Females ≥ 30 years old	C	1 (6-8)
Males ≥ 30 years old	D	1 (6-8)
Adult group specified by local PI, according to local context	Unspecified	1 (6-8)
Adult group specified by local PI, according to local context	Unspecified	1 (6-8)
Total		6 (36-48)

FGD: focus group discussion. ^*^Four different geographical areas within the HDSS (i.e four different communes) will be selected to conduct the first 4 preparatory focus groups discussion.

***Community members for focus group discussions.*** Community members 18 years old or older who are willing and able to express themselves in a group are eligible to participate in this component. In each study site, six to eight community members will be randomly recruited for each of the six preparatory FGDs, as identified in the HDSS database. Participants should all come from different households. Because the FGDs should represent community norms relating to demand, antibiotic suppliers and healthcare workers will be excluded from participation. Each focus group should be sufficiently homogeneous in gender and age in order to avoid a FGD being dominated by certain category of individuals.

The first four preparatory focus groups are stratified by sex and age according to Table 1, and will take place in four different geographical areas (i.e. neighborhoods) in the HDSS. All participants in a FGD should live in the same geographical area where the FGD is held.

Selection of participants in the remaining two focus groups will be based on social characteristics relevant to the local context, such as the age of the mother and children, or having older family members.

The rationale for this selection is similar to the inclusion criteria for choosing community members for IDIs as described above. Community members who participate in a FGD cannot participate in other parts of the study. However, any community member who does not consent to participate in a FGD will still be eligible for inclusion in an in-depth interview. If the final explanatory FGDs (Phase 4) require participants from outside the groups mentioned above, the inclusion criteria will be redefined for that site.

***Households for longitudinal survey.*** Households that already participate in the HDSS and have previously provided consent, are eligible to participate in this add-on study at two consecutive time points. An adult household representative (18 years or older) must provide additional written informed consent before the household can be included in the current add-on study. In each study site, 1,000 households will be recruited to participate in this add-on study.

***Customers for exit interview.*** At the selected antibiotic suppliers, each customer who leaves the drug outlet will be approached, and asked whether antibiotics were supplied to him/her. In case of an antibiotic encounter, the customer will be asked to participate in the customer exit interview. Recruitment of participants will be stopped whatever comes first, the end of that particular day or 30 antibiotic encounters.

To be included in the study, customers have to accept disclosing whether antibiotics were supplied to them and to consent to participate in an interview. Refusal cases will be registered.

### Sample size calculation

***Sample size HDSS household survey.*** This is the first study that will compare determinants of antibiotic use among LMICs through Sen’s Capability Approach. The main study outcome is the appropriate use of antibiotics and its determinants. In this study, the provision of antibiotics is considered to be appropriate if all of the following conditions are met: presence of original packaging, before expiration date, supplied with prescription, and with proper indications for use ^9, 10^. If any of these conditions is not met, the provision of antibiotics is considered to be inappropriate. This will be assessed by logistic regression analysis in each study site. In previous studies, antibiotics were supplied to outpatients with a prescription, which is an important criterion in our definition of appropriate use, in 60% of cases in Ghana ^11^, in 50% of cases in Bangladesh ^12^, but only in 10% of cases in Vietnam ^4^. Antibiotics were appropriately dispensed in 20% of pharmacies in Thailand, under criteria stricter than applied in the current study ^13^. Therefore, it is expected that on average 30% of antibiotic use will be classified as appropriate. Based on previous experience with surveys in the participating study areas, a loss to follow-up of 5–10% is to be expected. To avoid the need for subsequent recruitment of extra ‘replacement’ households, 1,100 households will initially be recruited (~4,400 household members), of which we expect at least 1,000 to participate in two consecutive surveys. Despite the use of show-cards, probably not all household interviewees will be informed about the antibiotic consumption by the household. If 750 HDSS household representatives (representing 3000 HDSS household members) can fully participate, and 20% of household members have an antibiotic intake in the months of survey, we may expect 180 appropriate antibiotic events. Given the rule of thumb of 10 events for each variable in regression analysis, 180 events would allow for 18 variables. This corresponds with the expected number of possible conversion factors to be yielded from the household questionnaires.

***Sample size customer exit survey.*** This study will compare antibiotic supply between LMICs using a standardized method. Therefore, the sample size is based on WHO recommendations ^14^ and on previous experience from customer exit interviews.

According to the WHO/INRUD methodology for investigating drug use in health facilities, a representative sample requires 30 prescribing encounters per facility from 20 facilities ^15^. A longitudinal study design is preferred, to account for potential seasonal variation in antibiotic supply ^16^. In addition, although the presence of interviewers could influence suppliers’ behaviour (Hawthorne effect), this effect is likely to diminish over time.

For each study site, 20 antibiotic suppliers will be selected for customer exit interviews. At each selected supplier, up to 30 antibiotic encounters will be observed on a single day of survey, amounting to a maximum of 600 antibiotic encounters per study site per round of survey. To account for seasonal variations, this survey will be performed at four time points (i.e. four days of survey) spread over a one-year period, synchronized across all study sites. In total, a maximum of 2,400 antibiotic encounters will be recorded at each study site.

The main study parameters are antibiotic exposure and antibiotic burden. Previous estimates of antibiotic exposure through customer exit surveys ranged from 21% at private retail pharmacies in India, 29% at drug stores in Mexico, to 39-43% at public/private suppliers in India ^17– 19^. Accounting for high antibiotic exposure of 43%, given an α of 0.05, the number of antibiotic encounters surveyed by this study will have a power of up to 0.90 to identify a significant difference in antibiotic exposure of 5% between study sites, and a power of up to 0.83 to identify a 10% difference between supplier types within each study site. Because the antibiotic burden will be calculated per type of antibiotic, and it is unknown to what extent different types of antibiotics will be supplied, no reliable power estimation can be given for this second parameter.

***Rationale for other sample sizes.*** The mapping of antibiotic suppliers will be as exhaustive as possible. The choice of a number of 10 inventories of antibiotic resources per study site is based on the expected minimum number of antibiotic suppliers present in any of the participating study sites. This sample will be sufficiently large to provide a fair representation of antibiotic suppliers. Qualitative investigation of this study topic is quite limited, but experience with qualitative research instruments in the participating study sites indicates that data saturation will be achieved with the number of interviews planned.

## Outcome measures

### Level of knowledge on antibiotics

To assess the level of a participant’s knowledge on antibiotics, the following three instruments will be employed in the HDSS survey and the antibiotic customer exit interview.

First, three multiple choice questions (each with four options) will be posed about antibiotics. Second, the HDSS field worker will display a first show-card with photos of three pills that are commonly available in the study area: paracetamol, a non-steroidal anti-inflammatory drug, and an antibiotic. For the development of these show-cards a pharmacist who works in the corresponding country and is knowledgeable about local outpatient antibiotic supplies will be consulted. The respondent will be asked to indicate the antibiotic pill. Third, the HDSS field worker will display a second show-card with photos of five different antibiotic pills that are commonly available in the study area and will confirm that these are in fact antibiotics.

If the respondent provides two or more correct answers to the multiple-choice questions, his/her level of knowledge on antibiotics is classified as A. If the respondent provides less than two correct answers to the multiple-choice question, but does correctly indicate the antibiotic pill on the first show-card, his/her level of knowledge on antibiotics is classified as B. If the respondent provides less than two correct answers to the multiple-choice question and does not correctly indicate the antibiotic pill on the first show-card, his/her level of knowledge on antibiotics is classified as C.

### Reported experiences

As mentioned above, the main study parameter of this study component is appropriate use of antibiotics, which is a composite measure. Conversion factors that may affect the relation between appropriate provision of antibiotics and appropriate use of antibiotics collected per study site are:

- Sex and age
- Education
- Socioeconomic status, according to metrics collected by the INDEPTH network HDSS surveillance site
- Level of knowledge on antibiotics: A / B / C
- The number of episodes of antibiotic intake per person during the months of survey
- The proportion of episodes of antibiotics intake that are preceded by a prescription
- The indications for antibiotics’ use as reported by the community members. The indications will be classified to one of the following categories, based upon the International Classification of Primary Care (ICPC-2e v5) ( http://www.kith.no/upload/2705/ICPC-2-English.pdf): chest pain / cough / dental / dyspnoea / ear and eye / fever / gastrointestinal / gynaecological / headache / male genital / musculoskeletal / nose / preventive / skin and soft tissue / surgery related / throat / urinary / wound.
- Susceptibility to infectious disease episodes expressed in vaccination status, presence of chronic diseases, hospitalization for more than one week in the past six months.

The exact classification for the following potential conversion factors that will be applied in the household survey, will be determined after their relevance and nature has been explored in the exploratory qualitative in-depth interviews and FGDs.

- Healthcare seeking behaviours
- Insurance coverage
- Sources of antibiotics (type of supplier, distance to, price)
- Knowledge of and attitudes towards healthcare, medicines, antibiotics and antibiotic resistance, of both antibiotic suppliers and users
- Expectations of and experiences with the local healthcare system
- Social and cultural context
- Regulatory issues

### Observed metrics

The main study parameters of this study component are the antibiotic exposure and the antibiotic burden within the community site studied. Antibiotic exposure is expressed as the proportion of customers leaving a supplier to whom antibiotics are supplied. To this end, the number of customer encounters with and without antibiotics will be recorded. Supplied antibiotics formulated in drops or creams are not considered to be an antibiotic encounter. The antibiotic burden is expressed in Defined Daily Doses (DDDs) supplied per 100 customer encounters on the day of survey, for each type of antibiotic. The DDD is the assumed average daily maintenance dose for a drug used for its main indication in adults ^20^. The denominator of antibiotic burden is introduced to account for the size of the customer population to which the DDDs are supplied. Here, the denominator is set to 100 customers encounters at the supplier involved on the day of survey. To enable calculation of antibiotic burden, the antibiotic’s name, strength, units and dose will be collected for each antibiotic encounter from the customer. Supplied antibiotics will be recorded by their generic names, and they will subsequently be classified according to the WHO Anatomical Therapeutic Chemical (ATC) classification system ^20^.

Additional parameters collected in each study site are:

- The distribution of supplier types: Suppliers will be classified by legal permit (yes/no), funding (public / private / missionary), and type (hospital pharmacy / retail pharmacy / clinical with physician / clinic without physician / chemical shop or drug store / convenience store or grocer / street or market vendor / community health worker / traditional healer). In addition, the number of daily antibiotic encounters will be estimated.
- The proportion of essential antibiotics that is available and appropriately provided, and their price: Appropriateness of provision will be assessed by storage conditions, original packaging, expiration date, appropriate package insert, presence of prescription and by verbal directions for use.
  The proportion of antibiotics that is supplied upon prescription.
- The distribution of customer types (age and sex) and end user types (a family member ≤ 17 years old / a family member ≥ 18 years old / a friend or relative / an animal / unspecified).
- Customers’ level of knowledge on antibiotics: A / B / C.
- The indications for use of antibiotics as reported by the customers: The indications will be classified to one of the following categories, based upon the International Classification of Primary Care (ICPC-2e v5): chest pain / cough / dental / dyspnoea / ear and eye / fever / gastrointestinal / gynaecological / headache / male genital / musculoskeletal / nose / preventive / skin and soft tissue / surgery related / throat / urinary / wound / other.

## Data analysis

Data analyses will be performed at several stages throughout the study. To facilitate uniform data processing, prior to the interviews, a workshop on data analysis will be provided to qualified representatives from each study site. Directly after the mapping of all antibiotic suppliers in a study region is completed (month 6), the rank in number of daily antibiotic encounters and the proportion of each type of antibiotic supplier will be calculated to inform the selection of supplier participants. Alongside the execution of the exploratory qualitative in-depth interviews and FGDs, the qualitative data will already be analyzed locally (month 7 to 10), thereby providing a basis for iteratively refining the design of upcoming interviews, and eventually the design of the quantitative HDSS household surveys. Soon after the first 6 months of the HDSS household survey and customer exit survey have passed (month 18), an interim analysis will be performed. These results will be triangulated with previously obtained qualitative data to design a second round of site-specific in-depth interviews and FGDs. The final seven months of the study will be dedicated to final data processing, analysis, and reporting.

### Reported experiences

To analyse the qualitative data, a thematic analytical approach will be employed ^5^. Verification of the data and themes will be conducted by members of the research team with skills in qualitative analysis. This will involve validating and assessing the trustworthiness of the qualitative data using the four constructs of credibility, transferability, dependability and conformability. The qualitative work will comply with the consolidated criteria for reporting qualitative research ( COREQ) ^6^.

The main quantitative parameters will be presented as a comparison between the six study sites. Appropriate use of antibiotics will be presented as a percentage of the reported episodes of antibiotic intake per study site. The relation between appropriate provision (also in % of antibiotic intake episodes) and appropriate use of antibiotics will be presented in a dot plot with each dot representing one study site. Finally, a model will present for each study site that contains the site-specific determinants of appropriate antibiotic use, accompanied by their odds ratios and 95% confidence intervals.

Statistical significance of differences in the proportion of antibiotic intake episodes that involves appropriate antibiotic use between study sites, will be tested by Chi square testing using the sum of all registries in each study site. The presence of a statistically significant correlation between appropriate antibiotic provision and appropriate antibiotic use across the six study sites will be assessed using Spearman rank testing. Potential determinants of appropriate antibiotic use will be identified by univariate binary regression analysis. Variables that display an association with appropriate antibiotic use in univariate analysis with a p-value < 0.1 will be selected for multivariate analysis (expected number 18, see paragraph *Sample size HDSS household survey*). True independent determinants of appropriate antibiotic use will be determined by multivariate binary logistic regression analysis using a stepwise backward elimination approach based on the probability of the likelihood-ratio statistic. All tests will be performed in two-sided fashion, and a p-value of <0.05 will be considered statistically significant.

### Observed metrics

The main study parameters will be presented as a comparison between the above listed seven study sites in six LMICs countries. Antibiotic exposure will simply be presented as the percentage per study site, derived from cumulative customer encounters surveyed at each study site (20 suppliers combined). The antibiotic burden needs to be normalized through calculation, prior to presentation in a summary metric per study site. First, the amount of antibiotics supplied is normalized to the number of Defined Daily Doses (DDDs), according to WHO guidance. Subsequently, the sum of DDDs supplied during the surveyed time frame is normalized for the size of the customer population to which the antibiotics were supplied in that time frame. Our standardized measure of antibiotic burden is the number of DDDs of a given antibiotic per 100 customers. This number will be obtained by taking the sum of DDDs at each supplier (four survey days combined), divided by the total number customer encounters surveyed, and multiplied by 100. The antibiotic burden is a continuous variable that will be presented as an average or median of its distribution among suppliers for each study site (depending on normality of data).

Missing data will be coded as such and accounted for in subsequent data representation and statistical analysis.

The data format for statistical testing of differences between study sites matches the data presentation as proposed above. For antibiotic exposure, the sum of all registries in each study site (from 20 suppliers combined) will be compared. For antibiotic burden, its distribution among surveyed suppliers per study site will be compared. Chi square testing will be applied for antibiotic exposure (Fisher exact if <10 cases in any cell), and for antibiotic burden a two-way ANOVA or Kruskal-Wallis test will be performed (depending on normality of data) for each type of antibiotics. All tests will be performed in two-sided fashion, and a p-value <0.05 will be considered statistically significant.

### Dissemination of outcomes

Data from this study will be reported and interpreted at a closing meeting. Subsequently, the results will be disseminated via meetings with stakeholders, conferences, and publications in peer reviewed journals. Authorship and reporting of this work will follow international guidelines.

Given that written informed consent is obtained for all study elements, the anonymized data collected in all study elements are eligible for use in future non-related studies.

## Conclusion

Currently, the study is ongoing at its third phase and will be completed by the end of 2018. The insights gained in relation to antibiotic practices in different LMIC will inform tailored intervention strategies to promote appropriate antibiotic use. In addition, this project provides a uniform framework for appraising current antibiotic use patterns, which may subsequently be used in other LMIC communities.

## Ethical statement

The protocol was reviewed by the Oxford University Tropical Research Ethics Committee (OxTREC, Reference: 31-15), and the local ethical committees of each participating study site including: (1) Vietnam Ministry of Health (MoH) Institutional Review Board (IRB) (6670/QD-BYT); (2) Institute for Population and Social Research (IPSR)-IRB, Mahidol University, Thailand (2016/04-035); (3) Ethical Review Committee (ERC), icddr,b, Bangladesh (PR-16053); (4) Ghana Health Service Ethics Review Committee (Dodowa site, GHS-ERC 15/06/16; Kintampo site, GHS-ERC 04/05/16); (5) Mozambique Ministry of Health IRB (CIBS-CISM/101/2016); and (6) Human Research Ethics Committee, University of the Witwatersrand, South Africa (M160753).

All potential participants will be informed about the study and asked for their consent by trained HDSS field workers. Information about the study will be provided both orally and in writing.

Given the real-time design of the customer exit interviews, potential participants will be informed about the study and asked for written consent during the course of one conversation. The local language written study information and informed consent criteria will be read aloud to a potential participant by the HDSS field worker. In addition, customer participants will receive a copy of the written study information. Only after written consent is provided will the interviews be conducted.

## Supplementary material

Study documents antibiotic suppliers:

- Supplementary File 1: eCRF registry form supplier characteristics.
- Supplementary File 2: eCRF supplier inventory.
- Supplementary File 3: Preparatory supplier in-depth interview guide.

Study documents community members:

- Supplementary File 4: Preparatory community member in-depth interview guide.
- Supplementary File 5: eCRF antibiotic knowledge community member in-depth interview.
- Supplementary File 6: Preparatory community member FGD guide.

Study documents HDSS households:

- Supplementary File 7: eCRF HDSS household member questionnaire.

Study documents exiting customers:

- Supplementary File 8: eCRF customer antibiotic encounter questionnaire.
