# Supplementary material for: Community-level antibiotic access and use (ABACUS) in low- and middle-income countries: Finding targets for social interventions to improve appropriate antimicrobial use – an observational multi-centre study
Source: Wellcome Open Res. 2017 Jul 28;2:58. [Version 1] doi: 10.12688/wellcomeopenres.11985.1 (PMC5897850; doi:10.12688/wellcomeopenres.11985.1)
Supplement: Supplementary file 1 [file wellcomeopenres-2-12958-s0000.tgz › 5a5b66cb-fa3c-4eaa-96b4-8bc11e02d7e3.docx]

**Supplementary File 1: eCRF registry form_supplier characteristics**

**Antibiotic supplier study code**: …………_............ [study site]_[antibiotic supplier]

Type: 🞎 Hospital pharmacy

🞎 Retail pharmacy

🞎 Clinic with physician

🞎 Clinic without physician

🞎 Chemical shop / Drug store

🞎 Convenience store / Grocer

🞎 Traditional healer

🞎 Community health worker

🞎 Street vendor (peddler) / Market vendor

🞎 Other: ……………………………………………………………………………...

Funding: 🞎 Public 🞎 Private 🞎 Missionary 🞎 Unknown

Legal / formal: 🞎 Yes 🞎 No 🞎 Unknown

Expected number of daily antibiotic

encounters: ……………………….

Moving location: 🞎 Yes 🞎 No

If moving location, contact information: ………………………………………………….............................................

If fixed location, GPS: ………………………, …………………………
