# Supplementary material for: Community-level antibiotic access and use (ABACUS) in low- and middle-income countries: Finding targets for social interventions to improve appropriate antimicrobial use – an observational multi-centre study
Source: Wellcome Open Res. 2017 Jul 28;2:58. [Version 1] doi: 10.12688/wellcomeopenres.11985.1 (PMC5897850; doi:10.12688/wellcomeopenres.11985.1)
Supplement: Supplementary file 2 [file wellcomeopenres-2-12958-s0001.tgz › 2bbe4f7c-da3f-4583-bddd-2c211a7c34c8.docx]

**Supplementary File 2: eCRF_supplier inventory**

1. *This is an inventory of the availability and provision quality of 5 essential systemic antibiotics. The example given below concerns amoxicillin; one of the 5 antibiotics inventoried*
2. *Most of the questions could be answered through direct observation without questioning the supplier*
3. *If possible, the inventory should be conducted with a senior, or otherwise knowledgeable pharmacy dispenser*
4. *Tablets, intravenous and intramuscular antibiotics are assessed, not other formula like droplets or creams*
5. *For each antibiotic inventoried, the corresponding required storage conditions are processed into the questionnaire (see table below).*

| **Antibiotic (ATC code)** | **Required storage conditions** |
| --- | --- |
| 1. Amoxicillin (J01CA04) | At ≥15 and ≤25 degrees Celsius, protected from light and moisture. |
| 2. Amoxicillin-clavulanic acid (J01CR02) | Protected from light and moisture. |
| 3. Chloramphenicol (J01AA01) | At ≥2 and ≤8 degrees Celsius, protected from light. (Suspensions should be stored in a fridge to storage instructions given above, but tablets in blister packs at ≤ 25 degrees Celsius) |
| 4. Ciprofloxacin (J01MA02) | None. |
| 5. Sulfamethoxazole-trimethoprim (J01EE01) | At ≤24 degrees Celsius. |

**ANTIBIOTIC SUPPLIER**  …….…_.........

[study site]_[antibiotic supplier]

*Amoxicillin (ATC code: J01CA04)*

1. Is the medicine available for dispensing at the moment? yes / no

*If yes, answer question 2 to 11 for each available brand of the particular antibiotic separately.*

*If no, proceed to the next antibiotic.*

1. What is the temperature at the medicine’s actual storage location? *(measured with a digital thermometer. Note that If the supplier does not want the field worker to see the storage area, they should be requested to place the thermometer in the relevant part of the storage area themselves, in order to obtain a valid temperature reading. Readings should be taken in the main storage area, not in the part of the shop where they are put on display.)*  …………….°C
2. Is the medicine stored protected from light? yes / no
3. What is the humidity at the medicine’s actual storage area? (*Measurement to be taken using a simple hygrometer, or humidity monitoring device*) ……………%
4. Is the medicine packaged in its closed and original blister pack? yes / no

*If no, skip to question 8*

1. *If yes*, is the blister pack packaged in its closed and original box? yes / no
2. Is an expiration date printed on the box? yes / no
3. Is the medicine before its expiration date? yes / no
4. Is the medicine accompanied by a package insert? yes / no

*If no, skip to question 11*

1. *If yes,* is the text of the package insert written in the prevailing language? yes / no
2. Write down the following information about the available brands of the medicine sold by the supplier in the table below:

| **Medicine brand** | **Dose (***mg)* | **Quantity** | **Formulation (***capsule/tablet/suspension intravenous/intramuscular)* | **Price** *( local currency)* | **Country of Manufacture** |
| --- | --- | --- | --- | --- | --- |
|  |  |  |  |  |  |
|  |  |  |  |  |  |
|  |  |  |  |  |  |
|  |  |  |  |  |  |

***The following questions are about antibiotics sold by the supplier (Circle only one answer for each question)***

1. Are antibiotics supplied exclusively with a prescription? yes / no / variable
2. Are antibiotics supplied in their closed and original blister pack? yes / no / variable
3. Are blister packs supplied in their closed and original box? yes / no / variable
4. Are antibiotics supplied with written instructions for use? yes / no / variable
5. Are antibiotics supplied with verbal instructions for use? yes / no / variable

We have finished the inventory. Thank you for your participation.
