# Supplementary material for: Community-level antibiotic access and use (ABACUS) in low- and middle-income countries: Finding targets for social interventions to improve appropriate antimicrobial use – an observational multi-centre study
Source: Wellcome Open Res. 2017 Jul 28;2:58. [Version 1] doi: 10.12688/wellcomeopenres.11985.1 (PMC5897850; doi:10.12688/wellcomeopenres.11985.1)
Supplement: Supplementary file 3 [file wellcomeopenres-2-12958-s0002.tgz › 059f685d-7108-44cc-a70f-7ed4414989ce.docx]

**Supplementary File 3: Preparatory_supplier_in-depth interview guide**

We need to be sure that we include the full range of the different types of antibiotic suppliers in the sample (licensed and otherwise), as identified in the mapping exercise in each HDSS. We should also include in the sample employees who work directly with customers. If someone was identified as selling antibiotics in the mapping exercise, but now they claim they don’t (perhaps for fear of prosecution if they are selling without a license), the interview should *not* go ahead with this outlet.

In order to ensure that these interviews produce good insights, the interviewers will need to have a good background understanding of their country’s regulations on antibiotic sales. This will need to be included in their training.

Informant data to be collected: Age, sex, location and type of business, length of time in the business (total and at current business), position in business, qualification.

*The medicines [QUESTIONS 2-5: * ONLY FOR BUSINESS OWNERS OR IN-CHARGE HEALTH WORKERS]*

1. Please tell me briefly about the range of different medicines that you sell. Which are the popular ones?
2. * What is/are the source/s of the various medicines that you sell? Do your suppliers bring the medicines to your shop, or do you pick them up yourselves from your suppliers?
3. * What informs your decision to buy from the suppliers that you have mentioned? (Probe for details of quality, cost, credit facilities, packaging/box/container, incentives, etc.)
4. * What are your experiences with getting your supply of different medicines? Probe regarding availability/shortages etc., and specifically for antibiotics.
5. * Where do you think the medicines you sell are manufactured? What are your impressions about the quality of the medicines from different origins? Are certain origins more popular than others?
6. Do you feel you know enough about the different medicines that you sell (what they treat, side effects etc.), or are there certain medicines that you would like to know more about? What would be the best way for you to learn about these medicines?
7. Which medicines do you usually sell to people who have coughs and colds? What determines the length of the course that you supply?
8. What do you know about antibiotics? Explain. What proportion of all the medicines that you sell are antibiotics? Which are your five most commonly sold antibiotics?
9. For which illnesses do you usually sell antibiotics? What determines the choice of antibiotic?
10. How and where do you store your antibiotics?
11. Do you check the expiry dates on the antibiotics you sell? What do you do with medicines that have passed their expiry date?
12. Do customers ever bring back unused medicines? If so, which medicines are most commonly brought back; and what do you do with these?

*The customers*

1. Can you describe how the dispensing process works here, from when a customer arrives to when they leave with their medicines?
2. Do many of your customers ask for particular medicines without any prescription (*either* through self-medication, *or* on verbal recommendation of health workers)? Is there a certain sort of customer who does this (e.g. male, female, young, old), and what are the most common conditions that they treat on this basis? Do they ask specifically for antibiotics? If so, which type of antibiotic?
3. Do you think that medicine sellers in this community ever feel encouraged to sell their customers antibiotics that may not be needed? If so, is this encouragement related to pressure from the customers or from the suppliers? Do the sellers go ahead and sell the medicines in such situations, or not? Details.
4. Do you ever give any sort of information (verbal or written) to your customers about the antibiotics that you sell them? What information do you give? Do they ever ask questions? Examples. *[Probe: do you ever ask about allergies?]*
5. Do your customers ever voice any concerns about the various medicines you sell? Details?
6. Are you aware of any medicine sellers in this area who *either* sell incomplete doses of antibiotics *[probe: if, for example, a customer doesn’t have money for the whole dose. Or for any other reason.]*, *or* who sell more antibiotics than are needed *[probe: for future use]?* Are these practices generally seen as acceptable or not? Details.
7. Do you ever dispense a mixture of antibiotics combined with other medicines? If yes, can you give an example of such a mix that you sell?
8. Do you think that your customers sometimes fail to take their full course of antibiotic treatment? If so, why do you think this is?
9. Do you ask your customers if they have used antibiotics before and for what conditions/diseases, in particular when they are requesting a specific antibiotic?

*Antibiotic resistance*

1. Do you think you have a good understanding of what antibiotic resistance is, how it is caused, and what its implications are? Is there anything on this topic that you would like to know more about? Details.
2. What do you think would be the best way to inform people (both medicine sellers and the community) about proper antibiotic use and the dangers of antibiotic resistance?
3. Overall, what do you think should be done to improve appropriate antibiotic use and decrease antibiotic resistance in this country?

*Regulatory issues [* ONLY FOR BUSINESS OWNERS OR IN-CHARGE HEALTH WORKERS]*

1. * What are the regulations that you have to follow in order to sell antibiotics [probe regarding prescriptions]? What challenges, if any, do you face in following these regulations?
2. * Do you think that most sellers know the regulations regarding selling antibiotics? If so, do they always follow them? If not, why not? Do you know whether these regulations are being checked or audited by authorities?
3. * Are there any penalties for sellers who sell antibiotics without prescription? Details.
4. * Do you think current government regulations are sufficient to control inappropriate antibiotic use? If not, how could things be improved?
5. * FOR PRIVATE SECTOR SUPPLIERS ONLY: What would happen to your business if antibiotic sales declined due to closer compliance with regulations on dispensing antibiotics? Would this be a problem for you or your business? If yes, how? What would you suggest as a means of resolving this problem?

We have finished the interview. Thank you for your participation.
