# Supplementary material for: Community-level antibiotic access and use (ABACUS) in low- and middle-income countries: Finding targets for social interventions to improve appropriate antimicrobial use – an observational multi-centre study
Source: Wellcome Open Res. 2017 Jul 28;2:58. [Version 1] doi: 10.12688/wellcomeopenres.11985.1 (PMC5897850; doi:10.12688/wellcomeopenres.11985.1)
Supplement: Supplementary file 4 [file wellcomeopenres-2-12958-s0003.tgz › 7389cbff-a95c-4bde-bb84-bbb5cc331ba2.docx]

**Supplementary File 4: Preparatory_community member_in-depth interview guide**

Informants should represent ordinary community members selected randomly from the HDSS database, reflecting the full geographical range of the site. For the preparatory in-depth interviews samples from three different community groups will be included. From the HDSS-database we will select 8 mothers who care for children ≤ 5 years old. The other 8 participants should not be mothers of children ≤ 5 years old, and include 2 males and 2 females ≥18 and <60 years old, plus 2 males and 2 females ≥60 years old. All participants should come from different households.

Informant data: Age, sex, occupation, location/neighbourhood of residence, level of education, literacy, religion, ethnic/tribal affiliation, how many/who lives at the house, health insurance.

*Accessing treatment*

1. How would you rate your overall health?
2. What do you do when you have any of these conditions: [Probes: self-medication, health centre, pharmacy, traditional healer etc.]
   1. Fever
   2. Headache
   3. Cough/cold
   4. Diarrhea
   5. Wound or skin infection
   6. Severe illness
   7. A sick child

Why do you choose these particular healthcare options?

1. How long does it take you to get to the place/s where you usually go for treatment, and how much does it cost to get there (if transport is necessary)? Is this convenient, or would you prefer something easier to reach?
2. How do you cover the costs for treatment when you or a member of your family is unwell? How easy is it for you to cover these costs, and what do you do if you are unable to cover them.
3. What would you do if there is a particular medicine you are seeking but the supplier doesn’t have it? (Probe: buy an alternative medicine from the supplier, go somewhere else etc.)
4. Do you ever ask for or use medicines from other people who have similar illnesses to your own? Details (e.g. from whom, which medicines, for which illness, reasons, etc.)

*The supplier/seller of medicines*

1. Do you usually ask the supplier for a particular medicine, or do you usually let them decide which would be best for the illness you have?
2. Do you usually receive instructions (verbal or written) for using the medicines from the supplier? In general, do you think they are well informed about the medicines they sell, so that you would trust their word and want to follow their instructions? Details. (Probe: by type of suppliers)
3. Do you ever buy less medicine / fewer pills than recommended by your healthcare provider? *[Probe: do you sometimes not have enough money to buy the full course of treatment?]* Details.

*The medicines*

1. What sort of medicines, if any, do you have at home at the moment, and what are these medicines for?
2. Do you know what antibiotics are, and what sort of conditions they treat? Can you give any examples of antibiotics that you know about?
3. *Now assess the participant’s level of knowledge on antibiotics as follows, and register the participant’s responses in the separate eCRF. First, pose the 3 multiple choice questions about antibiotics. At each question, ask the participant to select one of the 4 options listed. Second, display the first showcard with photos of 3 pills that are commonly available in the study area: paracetamol, a non-steroidal anti-inflammatory medicine, and an antibiotic. Ask the respondent to indicate which is the antibiotic pill according to him/her. Third, display the second showcard with photos of 5 different antibiotic pills that are commonly available in the study area and confirm that these are in fact antibiotics. Ask the participant if he/she visually recognizes any of the antibiotics and, if so, if he/she ever obtained antibiotics. Make sure that the participant’s responses are registered in the separate eCRF.*
4. *If the participant does not visually recognize any of the antibiotics OR has never obtained antibiotics, skip to question 21. Otherwise, continue with question 13.For each photo ask*: Have you or anyone in your close family ever used this antibiotic? What condition/illness was it used for? Was it effective or not? Do you have a preference for an antibiotic for certain conditions? If so, based on what? Other details.
5. Do you think that antibiotics have any particular benefits or risks? *[Probes: side effects, antibiotic resistance].*
6. If you want to buy antibiotics, what do you do? Do you have to get a prescription from the doctor first, or can you just go directly and buy them at the pharmacy/supplier? *[Probe: can you give more details about the type of supplier you go to, or describe a recent encounter with a supplier?]*
7. Only if you are willing to, you may answer the following questions, but no answer is needed. Are any of the medicines that you have at home now antibiotics? If so, what illnesses were they bought as treatment for?
8. Have you ever bought more antibiotics than you needed at the time, for future use? Details.
9. Do you sometimes stop taking your antibiotics before your pills are finished? Details (how and why). *[Probes: side effects, not sick anymore]*.
10. If you ever have unfinished antibiotics, what do you do with them? *[Probes: do you keep them? Do you ever give unused doses to other people who have the same illness you had?]*
11. Are you aware of any special instructions that you need to know about when you take antibiotics? Details.
12. Have you ever heard of antibiotic resistance? Do you know what it is, how it’s caused, and what its implications are? Is there anything on this topic that you would like to know more about? Details.
13. Do you think that the medicines you buy are usually of good quality? Have you ever had any bad experiences with medicines because you thought they were not good quality? Details.
14. Do you know whether medicines have an expiry date? If you think so, can / do you usually check the expiry date of the medicines you use? What do you do if the medicines are past their expiry date?
15. In general, where and how do you learn about medicines? If you wanted to know more, where would you go? What would be the best source of information about medicines for you?

We have finished the interview. Thank you for your participation.

If you are interested, you may take along with you this leaflet which contains more information about antibiotics and antibiotic resistance. *Provide printed information in local language.*
