# Supplementary material for: Community-level antibiotic access and use (ABACUS) in low- and middle-income countries: Finding targets for social interventions to improve appropriate antimicrobial use – an observational multi-centre study
Source: Wellcome Open Res. 2017 Jul 28;2:58. [Version 1] doi: 10.12688/wellcomeopenres.11985.1 (PMC5897850; doi:10.12688/wellcomeopenres.11985.1)
Supplement: Supplementary file 5 [file wellcomeopenres-2-12958-s0004.tgz › 23f80215-8c2f-4495-98da-2a3b9bebca40.docx]

**Supplementary File 5: eCRF antibiotic knowledge_community member_in-depth interview**

Participant study code: …………_............ [study site]_[preparatory (PI) or explanatory (EI) in-depth interview participant]

Multiple choice questions (select one answer per question)

1. What do you think are antibiotics? 🞎 Painkiller

🞎 Drug against fatigue

🞎 Drug against high blood pressure

🞎 Drug against infection

2. What do you think antibiotics do? 🞎 Decrease blood pressure

🞎 Give energy

🞎 Kill bacteria

🞎 Stop pain

3. When do you think should antibiotics be taken? 🞎 Bladder infection

🞎 Muscle pain

🞎 Weakness

🞎 Headache

First showcard (select one answer)

Indicated as antibiotic pill by the participant: 🞎 Photo of paracetamol

🞎 Photo of non-steroid anti-inflammatory drug

🞎 Photo of antibiotic

Second showcard

1. Does the participant recognize any of the antibiotics? 🞎 Yes 🞎 No

2. *If yes,* has the participant ever obtained antibiotics? 🞎 Yes 🞎 No
