# Supplementary material for: Community-level antibiotic access and use (ABACUS) in low- and middle-income countries: Finding targets for social interventions to improve appropriate antimicrobial use – an observational multi-centre study
Source: Wellcome Open Res. 2017 Jul 28;2:58. [Version 1] doi: 10.12688/wellcomeopenres.11985.1 (PMC5897850; doi:10.12688/wellcomeopenres.11985.1)
Supplement: Supplementary file 7 [file wellcomeopenres-2-12958-s0006.tgz › 39a83c96-cd43-4989-ae60-e27100209488.docx]

**Supplementary File 7: HDSS household questionnaire**

- The first section of this questionnaire primarily concerns the household as a whole, and it has only a limited focus on the individuals within it. The second section of the questionnaire includes questions for individuals, concerning their own antibiotic use.
- The first time a household is surveyed, *all* questions in *both* sections will be asked. The second time a household is surveyed, *only* the second round of questions will be addressed. The interviewer will start the second survey with displaying the second show-card with photos of 5 commonly available antibiotics, and confirm that these are in fact antibiotics.
- The multiple choice questions will be posed as open questions to the HDSS household representative. Subsequently the interviewer will tick the most appropriate option/s.

**HDSS HOUSEHOLD**

[study site]_[extant HDSS-coding]_[household survey #] …….…_........._.........

1. Today’s date dd / mm / yyyy

*Demographics of household – data to be incorporated directly into RedCAP from the HDSS database*

1. Village of residence …………………..………………….
2. Neighbourhood of residence …………………..………………….
3. The household’s socio-economic status according to metrics already collected by the HDSS

…………………

1. Number of people living in the household (as per HDSS definition)? …………………
2. Sex and age of all the people who live in the household

…………………………………….

…………………………………….

…………………………………….

…………………………………….

…………………………………….

…………………………………….

…………………………………….

…………………………………….

…………………………………….

…………………………………….

*Respondent details*

1. Respondent’s Date of Birth dd / mm / yyyy
2. Respondent’s Sex Male / female
3. What is your position in the household in relation to the household head? Self / Husband / wife / child / grandparent / other: …………………..

*Household characteristics*

1. What is the occupation of the main breadwinner in the household?
2. What is the highest education level completed of all the people who live in this household?

(tick one; None / primary school / secondary school / high school / technical school / higher or university)

1. Do you consider yourself to be religious in this household? If yes, which religion?

Yes: …………………..…… / No

*Access to healthcare*

1. How does your household cover the costs for healthcare?

(tick all that apply; paid health insurance / free health insurance / out-of-pocket / other)

1. What sorts of healthcare options are available to the people living in your household?

(tick all that apply; Hospital (Sub-District / District / Provincial), government clinic, private clinic, mobile health clinic, health centre, community health care worker/provider, Community Based Health Planning and Services (CHPS), Community Health Services (CHS), home based carer, traditional healer, herbalist, chemical shop, Drug store, pharmacy, drug peddler, prayer camps, Polyclinic, other)

1. Which of these healthcare options do each of the members of your household mostly go to first if they are *mildly* ill; and why? [INSTRUCTIONS TO REDCAP CONSTRUCTORS: PLEASE INCLUDE A TABLE WITH A ROW FOR EACH HOUSEHOLD MEMBER, AND COLUMNS FOR (I) THE TYPE OF HEALTH CARE OPTION THEY GO TO, AND (II) THE REASON WHY]

(tick all that apply for each member identified in the household roster; Hospital (Sub-District / District / Provincial), government clinic, private clinic, mobile health clinic, health centre, community health care worker/provider, Community Based Health Planning and Services (CHPS), Community Health Services (CHS), home based carer, traditional healer, herbalist, chemical shop, Drug store, pharmacy, drug peddler, prayer camps, Polyclinic, other)

*For why*: tick all that apply; cost / distance / trust / habit / time / popularity / advertising / convenience / disease severity / age of sick person / other

1. Which of these healthcare options would each of the members of your household go to first if they were *severely* ill; and why? [INSTRUCTIONS TO REDCAP CONSTRUCTORS: PLEASE INCLUDE A TABLE WITH A ROW FOR EACH HOUSEHOLD MEMBER, AND COLUMNS FOR (I) THE TYPE OF HEALTH CARE OPTION THEY GO TO, AND (II) THE REASON WHY]

(tick all that apply for each member identified in the household roster; Hospital (Sub-District / District / Provincial), government clinic, private clinic, mobile health clinic, health centre, community health care worker/provider, Community Based Health Planning and Services (CHPS), Community Health Services (CHS), home based carer, traditional healer, herbalist, chemical shop, Drug store, pharmacy, drug peddler, prayer camps, Polyclinic, other)

*For why*: tick all that apply; cost / distance / trust / habit / time / popularity / advertising / convenience / disease severity / age of sick person / other

1. Think of your household’s *most frequently used* healthcare provider when answering this question and Questions 19 and 20. By what means do household members usually travel there?

(tick one: walk, bicycle, motorbike, car, taxi, bus, other specified, don’t know)

1. How much does it cost your household to get to this *most frequently used* source of healthcare?

Amount …………….……..……/ no cost/ Don’t know

1. How long does it usually take to get to this *most frequently used* source of healthcare from your household?

……………h ……………min/ don’t know

1. *If there are any children under the age of 5 in the household*: Think of the healthcare provider your household usually uses for any under-5 year old children living in the household. By what means does whoever is responsible for the child usually travel there when taking the child for care?

(tick one: walk, bicycle, motorbike, car, taxi, bus, other specified, don’t know)

1. How much does it cost your household to get to this primary source of healthcare for a child under the age of 5?

Amount …………….……..………………….……..……/ no cost / Don’t know

1. How long does it take your household to get to this primary source of healthcare for a child under the age of 5?

……………h ……………min/ Don’t know

*Drug supplier*

1. What type/s of drug supplier do the members of your household attend?

(tick all that apply; Hospital (Sub-District / District / Provincial), government clinic, private clinic, mobile health clinic, health centre, community health care worker/provider, Community Based Health Planning and Services (CHPS), Community Health Services (CHS), home based carer, traditional healer, herbalist, chemical shop, Drug store, pharmacy, drug peddler, prayer camps, Polyclinic, other)

1. Which of the following factors determines the choices of drug supplier?

(tick all that apply; cost / distance / trust / habit / time / popularity / advertising / convenience / disease severity / age of sick person / other)

1. Do members of your household usually attend the same drug supplier? Yes / no / unknown
2. How often do your drug suppliers provide members of your household (including you) instructions for using the medicines they sell?

Always/Sometimes/Rarely/Never/Don’t know

1. How often do members of your household (including you) ask your drug suppliers questions about how to use medicines?

Always/Sometimes/Rarely/Never/Don’t know

1. How often have your drug suppliers given members of your household (including you) fewer medicines than were needed?

Always/Sometimes/Rarely/Never/Don’t know

1. How often have your drug suppliers given members of your household (including you) more medicines than were needed?

Always/Sometimes/Rarely/Never/Don’t know

*Sources of knowledge and awareness of household*

1. From where does your household get its information about medicines?

(tick all that apply; Health facility, hospital, TV, Radio, clinic, family members, neighbours, friends, poster at the clinic, pamphlet, leaflet, social media, internet, book, pharmacy, drug dispenser, other)

1. Which of the following types of medicine do you think is an antibiotic?

(select one; painkiller / drug against fatigue / drug against high blood pressure / drug against infection / other)

1. What do you think antibiotics do?

(select one; decrease blood pressure / give energy / kill bacteria / stop pain / stomach ache)

1. When do you think should antibiotics be taken?

(select one; bladder infection / muscle pain / weakness / stomach ache)

*Instruction for interviewer*: now display the first show-card with photos of three pills that are commonly available in the study area: paracetamol, a non-steroidal anti-inflammatory drug, and an antibiotic.

1. Which one pill do you think is an antibiotic? (select one; photo of paracetamol / photo of non-steroidal anti-inflammatory drug / photo of antibiotic)

We have finished this first part of the questionnaire.

**>>> SECOND SURVEY TO BE STARTED FROM THIS POINT <<<**

This section of the survey is about antibiotics. Antibiotics are medicines that are used to treat or prevent some types of infection. They work by killing the germs that cause the infections, or by preventing them from reproducing and spreading.

*Instruction for interviewer: now display the second show-card with photos of 5 different antibiotic pills that are commonly available in the study area and confirm with the respondent that these are in fact antibiotics.*

1. As far as you are aware, has anybody in this household taken antibiotics in the last month?

Yes / no / unknown

*If unknown, search for alternative adult household member who is informed about antibiotic consumption by the household. If no adult household member present is informed about the antibiotic consumption by the household, stop interview here.*

*If yes, antibiotics taken in the last month, complete questionnaire for* ***each individual household member*** *who has taken antibiotics.*

*If no, no antibiotics taken in the last month by any household member, stop interview here.*

*Recent antibiotic use* ***for each individual household member (up to 10 individuals)***. Adults will answer for themselves, guardians can answer on behalf of children.

1. Only if you are comfortable with it, I would like to ask for your permission to see the medicines you have in order to know if any antibiotics are present. If you are comfortable you can show these to me; if you are not comfortable, I will respect this and complete the questionnaire by asking you a few questions. *Permission provided?* Yes / no
2. *If permission is provided, are antibiotics identified by interviewer?* Yes / no
3. To what extent do you believe that your healthcare provider’s choice of medicines (antibiotics and other medicines) is best for you?

Always/Sometimes/Rarely/Never/Don’t know

1. Do you think that the medicines (antibiotics and other medicines) that you receive of good quality? Yes / no / it depends on the provider / unknown
2. What makes you prefer one medicine (antibiotics and other medicines) over another?

(tick all that apply; Cost, side effects, effectiveness, manufacturer, packaging, size of pill, colour, country of origin, brand, taste, expiry date, provider, ease of consumption, packaging, other)

1. Moving now specifically to antibiotics: Antibiotics are usually provided as a set of pills that are all supposed to be taken within a certain period – this set of pills is called a *course*. How many courses of antibiotics (which could include antibiotics that were previously obtained) have you taken in the last month? ………………….
2. For how many of the courses of antibiotics did you receive a prescription from a health worker ………………….
3. From what type of drug supplier did you receive the antibiotics?

(tick all that apply; Hospital (Sub-District / District / Provincial), government clinic, private clinic, mobile health clinic, health centre, community health care worker/provider, Community Based Health Planning and Services (CHPS), Community Health Services (CHS), home based carer, traditional healer, herbalist, chemical shop, Drug store, pharmacy, drug peddler, prayer camps, Polyclinic, other)

1. What are the names of the antibiotics you have taken in the last month?

………………… / Don’t know

1. Only if you are willing to, you may answer the following question, but no answer is needed. For what illness or illnesses did you take the antibiotics?

(Sore throat / cough / Flu / headache / Pain / Weakness / Wound / Dental / dyspnoea / ear / eye / nose / throat / fever / boil / gastrointestinal / Sexually Transmitted Infection / gynaecological / male genital / urinary tract infection / Chest pain / musculoskeletal / preventive / skin and soft tissue / surgery-related / HIV related opportunist infections / other (specify) / unknown.

1. How much did the antibiotics taken during the last month cost you? Amount ………………….

If more than one antibiotics course has been taken over the previous month, the questions below relate only to the most recent course.

1. Did you get a prescription for the antibiotics? Yes / no / unknown
2. Were the antibiotics supplied in a blister pack? Yes / no / unknown
3. *If no,* skip to Question 47. *If yes,* were the blister packs supplied in a closed box? Yes / no / unknown
4. Did you check if the antibiotic that you received had an expiration date written anywhere on the packaging/bag? Yes / no / unknown
5. *If no*, skip to Question 50. *If yes,* was there an expiration date written anywhere on the packaging/bag? Yes / no / unknown
6. *If no*, skip to Question 50. *If* *yes,* were the antibiotics supplied still before their expiration date? Yes / no / unknown
7. Did you receive any instructions for using the antibiotics?

(tick all that apply; Written / Oral / no / unknown)

1. *If no or unknown*, skip to Question 52. Did you follow these instructions? Yes / no / unknown
2. Did you obtain as many pills as your healthcare provider recommended or prescribed? Yes / no / unknown
3. *If you have finished the course*, did you take all the pills that you received?

Yes / no / unknown

1. *If you are still taking the course*, have you taken them all so far? Yes / no / unknown
2. How do you rate your overall experience with the drug supplier attended? Visual analogue scale
3. *If the answer to either of Questions 53 and 54 were ‘Yes’, skip this question*. Did you share your antibiotics with anyone else? Yes / no / unknown
4. *If the answer to either of Questions 53 and 54 were ‘Yes’, skip this question* Did you save any of the pills for future use? Yes / no / unknown

We have finished the questionnaire. Thank you for your participation.
