# Supplementary material for: Community-level antibiotic access and use (ABACUS) in low- and middle-income countries: Finding targets for social interventions to improve appropriate antimicrobial use – an observational multi-centre study
Source: Wellcome Open Res. 2017 Jul 28;2:58. [Version 1] doi: 10.12688/wellcomeopenres.11985.1 (PMC5897850; doi:10.12688/wellcomeopenres.11985.1)
Supplement: Supplementary file 8 [file wellcomeopenres-2-12958-s0007.tgz › a7573d1b-d52d-4c1d-8a5b-b7eb56b51786.docx]

**Supplementary File 8: eCRF_** **antibiotic encounter customer exit questionnaire**

i. The following questions will be answered for each type of antibiotic supplied to a particular customer.

ii. Tablets, capsules, paediatric formulations, as well as intravenous and intramuscular antibiotics are to be assessed, but not other formulae like droplets or creams.

iii. The reason-for-encounter symptoms are based on the International Classification of Primary Care (ICPC-2e v5 May 2015).

iv. As a matter of courtesy, the antibiotic supplier/dispenser should be informed that exit interviews will be conducted outside their facility.

**CUSTOMER EXIT INTERVIEW PARTICIPANT** …….…_........._.........

[study site]_[antibiotic supplier]_[customer exit interview participant]

*Demographics*

1. Age ………………………………years
2. Sex male / female
3. Only if you are comfortable with it, I would like to ask for your permission to examine your antibiotic/s together with you. If you are not comfortable with this, I will respect this and complete the interview without inspection of your antibiotics.

*Permission provided?* yes / no

*Antibiotics*

1. What is the name of the antibiotic/s you receive (Generic name, not brand name)? ……………………………………….
2. What is the size of one tablet? …………………mg / unknown
3. How many tablets are supplied? ……………………………………….
4. For how many days is this antibiotic supplied? ………………days / unknown
5. Were these antibiotics prescribed by a health professional? Yes / no
6. For who is this antibiotic?

(tick one; Myself/ Child family member / adult family member / friend or relative / animal / unspecified / other: …../ unknown)

1. Are you comfortable with telling us about the illness for which you got this antibiotic?

Yes / no

*If no, skip to Question 12*

1. *If yes,* for what illness did you receive this antibiotic? Sore throat / cough / Flu / headache / Pain / Weakness / Wound / Dental / dyspnoea / ear / eye / nose / throat / fever / boil / gastrointestinal / Sexually Transmitted Infection / gynaecological / male genital / urinary tract infection / Chest pain / musculoskeletal / preventive / skin and soft tissue / surgery-related / HIV related opportunist infections / other (specify) / unknown.
2. Today is [*select one option*] the expiry date. before / after / unknown
3. Did you receive written instructions for use? yes / no
4. Did you receive verbal instructions for use? yes / no
5. How do you rate your overall experience with the medicine supplier attended?

Visual analogue scale

We have finished the interview. Thank you for your participation.
