# Supplementary material for: Community-level antibiotic access and use (ABACUS) in low- and middle-income countries: Finding targets for social interventions to improve appropriate antimicrobial use – an observational multi-centre study
Source: Wellcome Open Res. 2017 Jul 28;2:58. [Version 1] doi: 10.12688/wellcomeopenres.11985.1 (PMC5897850; doi:10.12688/wellcomeopenres.11985.1)
Supplement: Peer review history

# Peer review: Referee response for version 1

**DOI:** 10.21956/wellcomeopenres.12958.r26565

**Published:** 2017-10-25

**Authors:** Hsu Li Yang

**Affiliations:** 1 Saw Swee Hock School of Public Health, National University of Singapore, Singapore, Singapore

This is a detailed and well-written protocol paper, the aim of the study being to investigate community antibiotic use in 6 LMICs in Africa and Asia. It seems like a very complex study to coordinate, hence the use and collaboration of the INDEPTH network. That also seems to be the limitation - it is not clear if INDEPTH network sites in these countries differ from those sites where INDEPTH does not operate as many of these are large countries where a variety of antibiotic supply and usage practices may exist in different geographic locations.

 Nonetheless, this study will advance our knowledge of antibiotic prescription and usage practices in the 6 countries, and I look forward to learning about the results.

I have read this submission. I believe that I have an appropriate level of expertise to confirm that it is of an acceptable scientific standard.

# Peer review: Referee response for version 1

**DOI:** 10.21956/wellcomeopenres.12958.r25401

**Published:** 2017-09-19

**Authors:** Iruka N. Okeke

**Affiliations:** 1 Department of Pharmaceutical Microbiology, Faculty of Pharmacy, University of Ibadan, Oyo State, Nigeria

Datasets are not relevant to this paper: it is a protocol.

 This paper describes a protocol that will use six sites in the INDEPTH network to study antimicrobial access and use in resource limited settings. Antibiotic use and distribution patterns will be catalogued following interviews and focus group discussions with suppliers and patients. A longitudinal survey will also be nested in the HDSS questionnaire. Thereafter use will be monitored by mapping and inventorying supply chains.  The study will further investigate discrepancies between use data reported in qualitative surveys and inventoried amounts of antibiotic.

 Antibiotic use is poorly understood in most non-affluent countries where availability without prescriptions and the existence of robust informal sectors for their distribution pervade. Therefore this is a valuable study that will be of interest to readers who work in or on this type of setting.

 The study hinges on its good design and conducting the right number and types of interviews in order to garner a realistic picture of antimicrobial use. I found the design adequate for this purpose and especially liked some of its iterative components, which ensure that preconceived ideas, which have a high likelihood of being wrong in this little-studied field, are not the basis for designing key tools for the study. Performing the study at HDSSes helps to ensure appropriate coverage and provide important background information. Additionally, extracting use information using a variety of research methods as the authors plan to do, has the best chance of uncovering unknown or under-recognized facets of antimicrobial use. Given that I am a scientist, I would also suggest social scientist review the methodological components of the protocol as well. 

 My one critique is connected to a theoretical base for the study. My familiarity with Amartya Sen's Capability Approach is limited but I found it difficult to map onto the antimicrobial use and access problem that the investigators are studying. Unlike other resources that are more directly connected to wealth, needing or using more antibiotics is not necessarily a reflection of wealth or better resources. Improving access is good but only to certain extents and within certain parameters. And the gold standard for antibiotic use would be prescription after susceptibility pattern but access to testing and prescribers are not part of this study. There are economic analogies to the antimicrobial use and resistance paradigms (for example, antimicrobial susceptibility is a resource that has been over-exploited in a manner that many common-use resources have), I am just not sure that this is the right one. I am not sure that this approach adds anything to the study, or if so, what the value of what it adds actually is. If there is value, this could be made clearer.

I have read this submission. I believe that I have an appropriate level of expertise to confirm that it is of an acceptable scientific standard.

# Response

**Published:** 2017-09-25

**Authors:** Nga Do

**Affiliations:** Oxford University Clinical Research Unit, Vietnam, Vietnam

Amartya Sen’s capability approach, although often used in relation to wealth, need not be limited to economic inputs. The framework allows a more nuanced approach to considering how various inputs do or do not translate to desired outcomes. A simple example to illustrate its utility is the case of food provision during famines. Simply providing food does not necessarily alleviate hunger during a famine. The Capability Approach allows us to understand how people translate the resource, in this case food, to a desired outcome, in this case alleviation from hunger. What we are arguing here with the Capability Approach is that ‘appropriate’ provision of antibiotics may not translate to its ‘appropriate’ use and the factors that affect this disconnect need to be examined. If we assume that antibiotics are prescribed appropriately, what are the factors that may influence their inappropriate use. Factors may include cultural norms, social practices, or economic constraints. Teasing out how and why people use antibiotics in ways that can lead to antibiotic resistance will help us develop targeted interventions based on evidence rather than assumptions.
